# Short-term inhalation of isoflurane improves the outcomes of intraportal hepatocyte transplantation

**DOI:** 10.1038/s41598-022-08237-6

**Published:** 2022-03-10

**Authors:** Hiroyasu Nishimaki, Yoshikatsu Saitoh, Akiko Inagaki, Yasuhiro Nakamura, Takehiro Imura, Ibrahim Fathi, Hiroki Yamana, Kazuo Ohashi, Shigehito Miyagi, Takashi Kamei, Michiaki Unno, Masafumi Goto

**Affiliations:** 1 Department of Surgery, Tohoku University Graduate School of Medicine, Sendai, 980-0872 Japan; 2 Division of Transplantation and Regenerative Medicine, Tohoku University Graduate School of Medicine, 980-0872 Sendai, Japan; 3 Division of Pathology, Faculty of Medicine, Tohoku Medical and Pharmaceutical University, Sendai, 983-8536 Japan; 4 Graduate School and School of Pharmaceutical Sciences, Osaka University, Osaka, 565-0871 Japan

**Keywords:** Cytokines, Hepatocytes

## Abstract

Clinical hepatocyte transplantation (HTx) is only performed without general anesthesia, while inhalation anesthetics are usually used in animal experiments. We hypothesized that isoflurane may be a possible reason for the discrepancy between the results of animal experiments and the clinical outcomes of HTx. Syngeneic rat hepatocytes (1.0 × 10^7^) were transplanted to analbuminemic rats with (ISO group) and without (AW group) isoflurane. The serum albumin, AST, ALT, LDH levels and several inflammatory mediators were analyzed. Immunohistochemical staining and ex vivo imaging were also performed. The serum albumin levels of the ISO group were significantly higher in comparison to the AW group (p < 0.05). The serum AST, ALT, LDH levels of the ISO group were significantly suppressed in comparison to the AW group (p < 0.0001, respectively). The serum IL-1β, IL-10, IL-18, MCP-1, RNTES, Fractalkine and LIX levels were significantly suppressed in the ISO group. The ischemic regions of the recipient livers in the ISO group tended to be smaller than the AW group; however, the distribution of transplanted hepatocytes in the liver parenchyma was comparable between the two groups. Isoflurane may at least in part be a reason for the discrepancy between the results of animal experiments and the clinical outcomes of HTx.

## Introduction

Liver transplantation is currently well-recognized as an established treatment for endo-stage liver disease^1^. However, this treatment is considered too invasive for patients suffering from acute liver failure and metabolic liver diseases^2–4^. Furthermore, the long waiting time to liver transplantation due to a shortage of organ donors^5^, has been pointed out as another severe obstacle to be overcome. Hepatocyte transplantation, in which isolated hepatocytes are infused to the recipient portal vein through a catheter, is less invasive in comparison to liver transplantation, since this approach requires no laparotomy at all, and because the whole procedure can be completed within a short period. Moreover, hepatocyte transplantation has the potential advantage of using fatty livers and cardiac arrest donor livers that are usually not suitable for liver transplantation^6^. Thus, hepatocyte transplantation is expected to serve as an alternative therapy to liver transplantation, especially for patients with metabolic liver diseases.

Hepatocyte transplantation has actually been applied in the clinical setting worldwide^7^. However, the outcomes of hepatocyte transplantation are still far from satisfactory^8–11^. Hepatocyte transplantation is obviously associated with many hurdles, including hepatocyte isolation^12^, graft preservation^13^, graft quality evaluation^14^, and hepatocyte engraftment^15,16^. Among these factors, the extremely poor engraftment of hepatocytes is a high-priority issue that must be overcome. Various animal models of hepatocyte transplantation have been used to solve this important issue. In contrast to the clinical situation, certain positive results of hepatocyte transplantation have actually been reported in animal experiments^16–21^, suggesting that there is a large discrepancy between the experimental results of animal models and clinical outcomes. It should be noted that clinical hepatocyte transplantation is only performed without general anesthesia, since this feature has been considered to be one of the most attractive advantages of hepatocyte transplantation. However, in animal experiments, it was necessary to use some type of general anesthesia during hepatocyte transplantation, due to technical restrictions and for the protection of animal welfare. Thus, no reports have demonstrated the direct effects of general anesthetics on the results of hepatocyte transplantation. Volatile inhalation gas is one of the most common anesthetics in animal hepatocyte transplantation experiments. Isoflurane, which is one of the most widely used volatile inhalation anesthetics in animal experiments, is known to have a strong vasodilatory effect^22,23^, portal pressure inhibitory effect^24,25^, and cytoprotective effect against cytokine-induced injury^26–28^. Of particular interest, Slehria et al. previously reported that the use of vasodilators dramatically increased the entry of transplanted hepatocytes into the recipient’s liver tissues, and improved hepatocyte engraftment^29^.

Thus, in the present study, we hypothesized that the use of volatile inhalation anesthetics, especially isoflurane, might be one explanation for the discrepancy between the results of animal experiments and the clinical outcomes of hepatocyte transplantation. In the present study, we developed a unique animal model for performing hepatocyte transplantation without general anesthesia. Using this model, we investigated the vasodilatory and anti-inflammatory effects of isoflurane on the intrahepatic distribution and engraftment of transplanted hepatocytes.

## Results

### Hepatocyte engraftment after transplantation with and without the short-term inhalation of isoflurane

**Figure 1:**
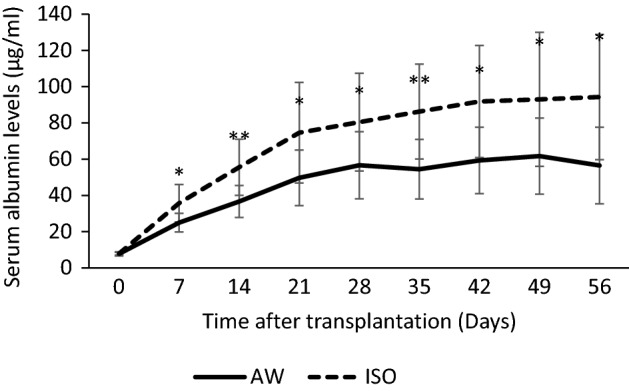
Hepatocyte engraftment after transplantation with and without the short-term inhalation of isoflurane. The serum albumin levels of the ISO group (dotted line, n = 10) were significantly higher than those of the AW group (solid line, n = 10) (*p < 0.05, **p < 0.01). The error bars represent standard deviation.

Hepatocyte engraftment was evaluated by measuring the serum albumin levels in the recipient. In the ISO group, the serum albumin levels gradually increased throughout the whole study period. In contrast, in the AW group, the serum albumin levels appeared to plateau at 28 days after hepatocyte transplantation. The serum albumin levels of the ISO group (pre-transplantation: 7.8 ± 0.8 μg/mL, day 7: 35.7 ± 10.4 μg/mL, day 14: 55.5 ± 15.4 μg/mL, day 21: 74.5 ± 27.8 μg/mL, day 28: 80.4 ± 26.9 μg/mL, day 35: 86.2 ± 26.2 μg/mL, day 42: 91.8 ± 30.9 μg/mL, day 49: 93.0 ± 37.0 μg/mL, day 56: 94.3 ± 34.6 μg/mL) were significantly higher in comparison to the AW group (pre-transplantation: 7.7 ± 1.1 μg/mL, day 7: 25.0 ± 5.2 μg/mL, p = 0.031, day 14: 36.6 ± 8.8 μg/mL, p = 0.004, day 21: 49.7 ± 15.4 μg/mL, p = 0.026, day 28: 56.6 ± 18.5 μg/mL, p = 0.031, day 35: 54.4 ± 16.4 μg/mL, p = 0.009, day 42: 59.3 ± 18.3 μg/mL, p = 0.025, day 49: 61.7 ± 21.0 μg/mL, p = 0.034, day 56: 56.5 ± 21.2 μg/mL, p = 0.014) in the present study (*p < 0.05, **p < 0.01) (Fig. 1).

### The AST, ALT, and LDH levels after hepatocyte transplantation with and without the short-term inhalation of isoflurane

**Figure 2:**
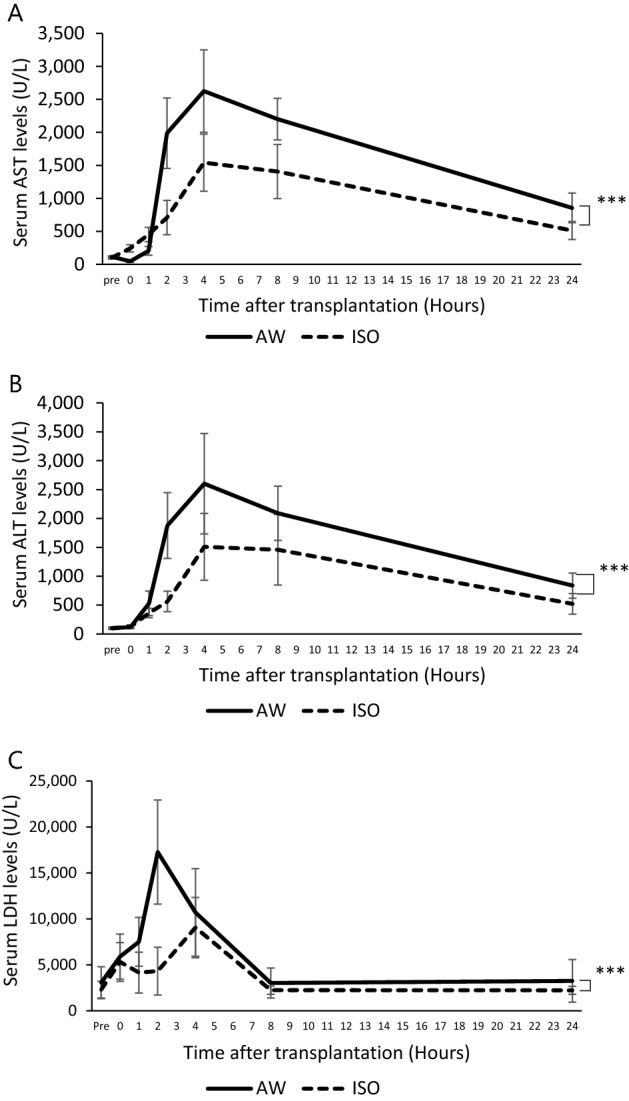
The AST, ALT, and LDH levels after hepatocyte transplantation with and without the short-term inhalation of isoflurane. (**A**) The serum AST levels of the ISO group (dotted line, n = 9) were significantly suppressed in comparison to the AW group (solid line, n = 10) (***p < 0.0001). (**B**) The serum ALT levels of the ISO group (dotted line, n = 9) were significantly suppressed in comparison to the AW group (solid line, n = 10) (***p < 0.0001). (**C**) The serum LDH levels of the ISO group (dotted line, n = 9) were significantly suppressed in comparison to the AW group (solid line, n = 10) (***p < 0.0001). The error bars represent standard deviation.

The serum AST levels of the ISO group (pre-catheter insertion: 102.0 ± 20.8 U/l, 0 h: 242.8 ± 54.5 U/L, 1 h: 453.2 ± 108.2 U/L, 2 h: 709.8 ± 259.0 U/L, 4 h: 1541.3 ± 433.2 U/L, 8 h: 1407.1 ± 409.5 U/L, 24 h: 512.0 ± 136.5 U/L) were significantly lower than those of the AW group (pre-catheter insertion: 115.2 ± 11.4 U/L, 0 h: 46.7 ± 54.5 U/L, 1 h: 204.9 ± 64.8 U/L, 2 h: 1988.0 ± 533.3 U/L, 4 h: 2625.2 ± 625.1 U/L, 8 h: 2200.8 ± 315.6 U/L, 24 h: 855.2 ± 224.6 U/L) in the present study (p < 0.0001) (Fig. 2A). The serum ALT levels of the ISO group (pre-catheter insertion: 97.2 ± 18.6 U/L, 0 h: 126.4 ± 31.6 U/L, 1 h: 362.4 ± 80.8 U/L, 2 h: 563.6 ± 178.0 U/L, 4 h: 1508.9 ± 578.5 U/L, 8 h: 1458.7 ± 611.9 U/L, 24 h: 521.8 ± 177.7 U/L) were also significantly suppressed in comparison to the AW group (pre-catheter insertion: 102.8 ± 19.0 U/L, 0 h: 118.4 ± 19.3 U/L, 1 h: 530.4 ± 212.2 U/L, 2 h: 1878.4 ± 568.8 U/L, 4 h: 2602.4 ± 868.8 U/L, 8 h: 2090.8 ± 470.4 U/L, 24 h: 838.4 ± 217.7 U/L) (p < 0.0001) (Fig. 2B). Likewise, the serum LDH levels of the ISO group (pre-catheter insertion: 2300.4 ± 915.9 U/L, 0 h: 5312.4 ± 2101.7 U/L, 1 h: 4152.0 ± 2217.5 U/L, 2 h: 4308.9 ± 2596.6 U/L, 4 h: 9054.7 ± 3285.4 U/L, 8 h: 2245.8 ± 453.0 U/L, 24 h: 2221.8 ± 428.3 U/L) were significantly lower than those of the AW group (pre-catheter insertion: 3054.8 ± 1743.0 U/L, 0 h: 5891.6 ± 2451.2 U/L, 1 h: 7502.8 ± 2654.1 U/L, 2 h: 17,268.0 ± 5666.3 U/L, 4 h: 10,697.6 ± 4768.1 U/L, 8 h: 3022.4 ± 1632.8 U/L, 24 h: 3746.4 ± 2318.8 U/L) during the whole study period (p < 0.0001) (Fig. 2C).

### Inflammatory mediators after hepatocyte transplantation with and without the short-term inhalation of isoflurane

**Figure 3:**
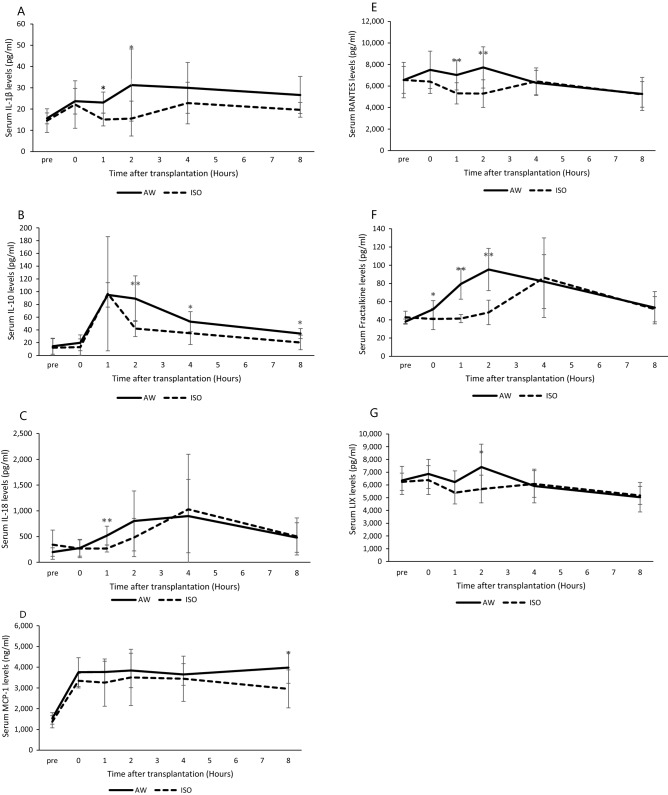
Inflammatory mediator levels after hepatocyte transplantation with and without the short-term inhalation of isoflurane. The serum levels of IL-1β (**A**), IL-10 (**B**), IL-18 (**C**), MCP-1 (**D**), RANTES (**E**), Fractalkine (**F**), and LIX (**G**) in the ISO group (dotted line, n = 9) were significantly downregulated in comparison to the AW group (solid line, n = 10) (*p < 0.05, **p < 0.01, ***p < 0.0001). The error bars represent standard deviation.

In order to examine the influence of isoflurane inhalation on inflammatory mediators in the recipients after hepatocyte transplantation, serum samples were analyzed using the Milliplex MAP Rat Cytokine/Chemokine Magnetic Bead Panel. As shown in Fig. 3A–G, the serum levels of IL-1β (p < 0.01), IL-10 (p < 0.01), IL-18 (p < 0.01), MCP-1 (p < 0.05), RANTES (p < 0.01), Fractalkine (p < 0.01), and LIX (p < 0.05) in the ISO group were significantly downregulated in comparison to the AW group. Notably, the serum levels of MCP-1, IP-10, RANTES, LIX, and Fractalkine in the ISO group were already suppressed at time 0 (before hepatocyte transplantation) in comparison to the AW group, suggesting that isoflurane may regulate the inflammatory status of liver tissues not only due to hepatocyte transplantation, but also due to catheter insertion into the portal vein.

### Immunohistochemical staining of the transplanted hepatocytes

**Figure 4:**
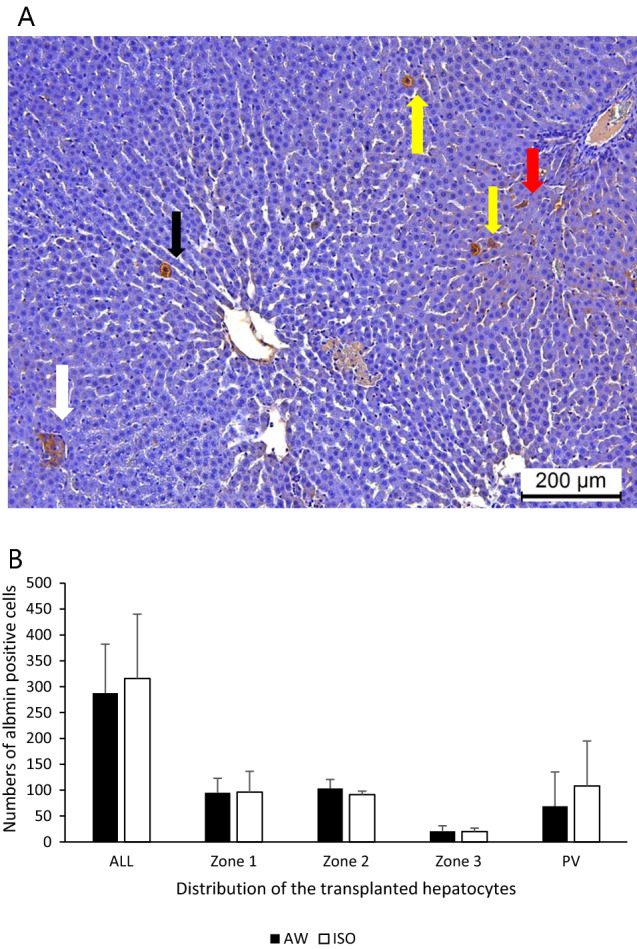
Albumin staining of the transplanted hepatocytes in the AW and ISO groups. (**A**) A representative photomicrograph of the albumin staining. The red arrow shows an albumin-positive hepatocyte in zone 1. Yellow arrows show albumin-positive hepatocytes in zone 2. The black arrow shows an albumin-positive hepatocyte in zone 3. The white arrow shows an albumin-positive hepatocyte in the portal vein (magnification: × 100, scale bar: 200 μm, *P* portal vein radicles, *CV* central vein). Albumin-positive hepatocyte grafts were widely distributed in the liver sinusoid in both groups. (**B**) The total numbers of albumin-positive hepatocyte grafts at 1 day after transplantation were comparable between the AW (black bar, n = 6) and ISO (white bar, n = 6) groups. Regarding the distribution of the transplanted hepatocytes in the recipient livers, no significant differences were observed between the two groups in any area. The error bars represent standard deviation.

The albumin-positive hepatocyte grafts showed a wide distribution in the liver sinusoid in both groups (Fig. 4A). The total numbers of albumin-positive hepatocyte grafts at 1 day after transplantation were comparable between the AW and ISO groups (Fig. 4B). Regarding the distribution of transplanted hepatocytes in recipient livers, no significant differences were observed between the two groups at zone 1 (AW: 95.0 ± 28.0 vs. ISO: 96.2 ± 40.4, p = 0.936), zone 2 (AW: 103.2 ± 17.4 vs. ISO: 91.5 ± 6.8, p = 0.388), zone 3 (AW: 20.5 ± 10.8 vs. ISO: 20.0 ± 6.8, p = 0.810), or the portal vein (AW: 68.8 ± 66.2 vs. ISO: 108.2 ± 87.0, p = 0.471).

### Ex vivo imaging evaluation of the transplanted hepatocytes

**Figure 5:**
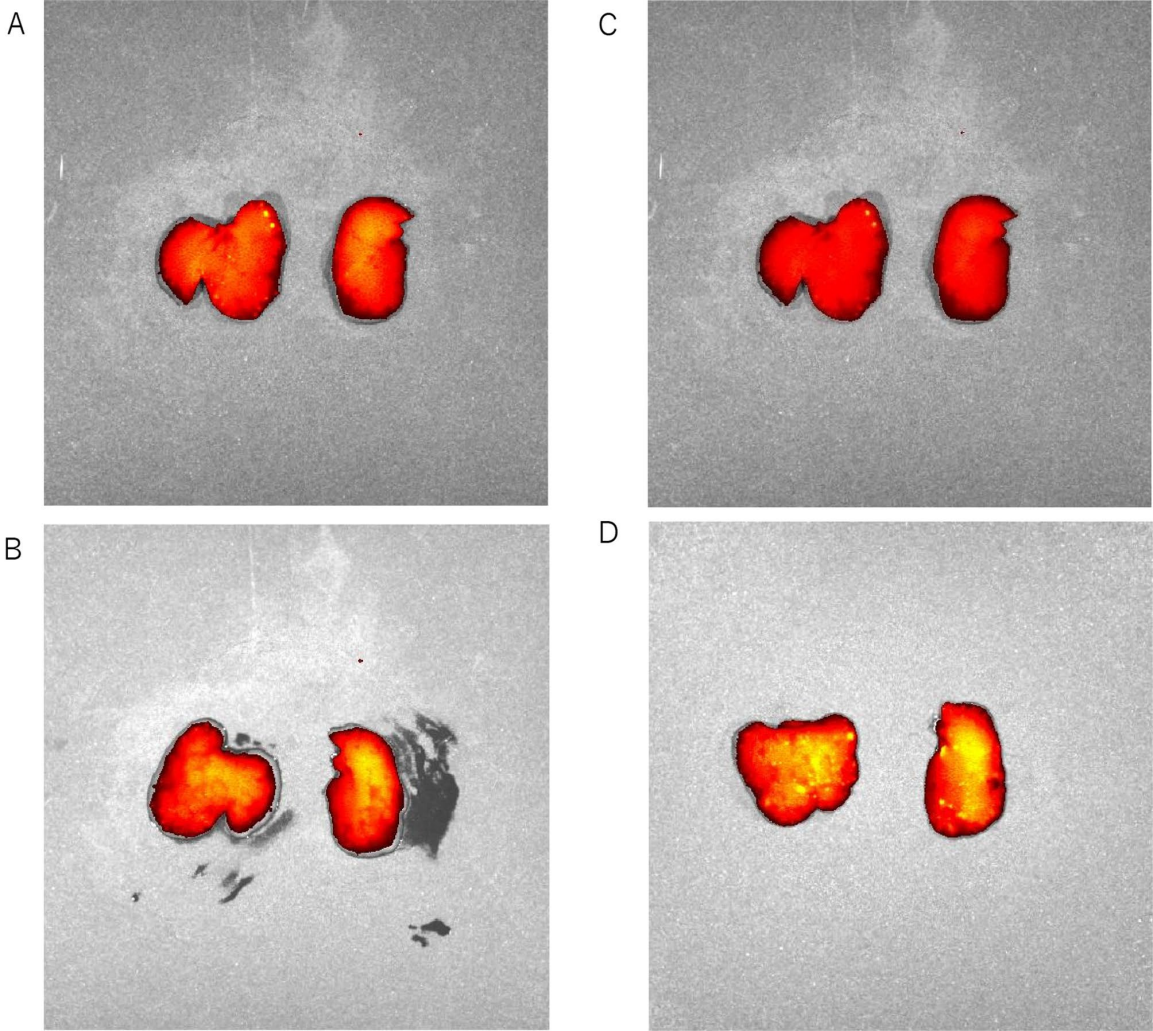
Ex vivo imaging evaluation of the transplanted hepatocytes. (**A**,**B**) A representative ex vivo image of the liver in the AW group (n = 3) at 1 day after transplantation (**A**: downward view of the liver, **B**: upward view of the liver). (**C**,**D**) A representative ex vivo image of the liver in the ISO group (n = 4) at 1 day after transplantation (**C**: downward view of the liver, **D**: upward view of the liver). The distribution pattern of the transplanted hepatocytes in the recipient livers appeared to be similar between the AW and ISO groups. The error bars represent standard deviation.

The hepatocytes transplanted via the portal vein catheter were only distributed in the liver (data not shown). The survival rate of the fluorescent signals at 1 day after transplantation was comparable between the AW (89.3 ± 10.8%) and ISO (74.0 ± 7.2%) groups. The distribution pattern of the transplanted hepatocytes in the recipient livers appeared to be similar between the AW and ISO groups (Fig. 5A–D).

### Evaluation of the ischemic liver tissue in the AW and ISO groups

**Figure 6:**
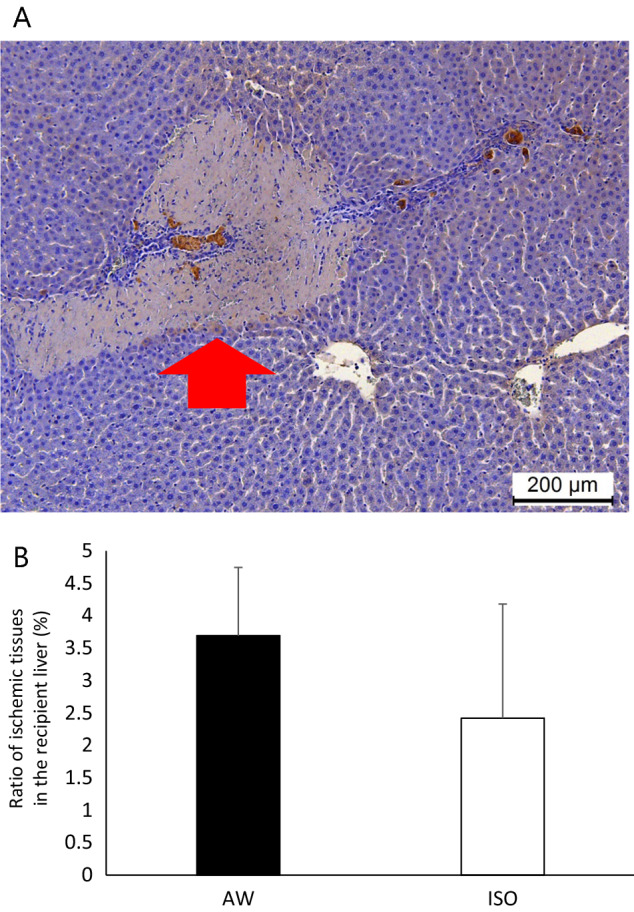
Evaluation of ischemic liver tissues in the AW and ISO groups. (**A**) A representative photomicrograph of the ischemic region (red arrow) in the liver parenchyma (magnification: × 100, scale bar: 200 μm). (**B**) The ischemic liver tissues tended to be more evident in the AW group (3.69 ± 1.05%, n = 6) in comparison to the ISO group (2.42 ± 1.76%, n = 6), although the difference did not reach statistical significance (p = 0.07). The error bars represent standard deviation.

The ischemic regions, which were easily detected in the livers of both groups (Fig. 6A), tended to be more evident in the AW group (3.69 ± 1.05%) in comparison to the ISO group (2.42 ± 1.76%), although the difference did not reach statistical significance (p = 0.078) (Fig. 6B).

## Discussion

In the present study, we demonstrated—for the first time—that short-term inhalation of isoflurane, which is one of the most popular and safe anesthetics, could improve the outcomes of intraportal hepatocyte transplantation. According to the detailed analyses in this study, the anti-inflammatory effects, rather than vasodilatory effects, of isoflurane appeared to be the main mechanism of the abovementioned benefits. Given that clinical hepatocyte transplantation is only performed without general anesthesia—unlike animal experiments in which it is only performed under general anesthesia—the large discrepancy between the results of animal experiments and the clinical outcomes of hepatocyte transplantation may logically be explained by the use of isoflurane.

This study clearly showed that several inflammatory mediators, including IL-1β, IL-10, IL-18, MCP-1, RANTES, Fractalkine, and LIX, were effectively suppressed by short-term inhalation of isoflurane in hepatocyte transplantation. In accordance with our findings, these beneficial anti-inflammatory effects of isoflurane have been widely reported in several fields other than hepatocyte transplantation^26,30–33^. In addition, Lee et al. recently reported that an alpha-1 antitrypsin improved hepatocyte engraftment by regulating IL-1β^34^. Taken together, these findings suggest that the anti-inflammatory effects of isoflurane may play an important role in promoting hepatocyte engraftment. On the other hand, the serum levels of IL-2, IL-5, IL-17, and IL-12p70 were upregulated in the ISO group in comparison to the AW group. Although IL-2 and IL-17 were previously reported to be induced by isoflurane^31,35^ and sevoflurane (a similar type of volatile inhalation anesthetic to isoflurane)^36^, in the present study, all values of abovementioned mediators were well controlled to below the pre-values (before catheter insertion) throughout the study period, irrespective of the study group. Therefore, the exact reason why the levels of the abovementioned mediators were higher in the ISO group remains unknown; however, it is most likely based on individual differences among recipient animals.

Unexpectedly, in this study, the distribution of transplanted hepatocytes in the liver parenchyma was comparable between the ISO and AW groups. Considering the previous studies that reported that vasodilators effectively guide transplanted hepatocytes into the deep areas of recipient liver sinusoids and improve hepatocyte engraftment^29,37^, the vasodilatory effects of isoflurane might have been insufficient in this study. Therefore, isoflurane combined with useful vasodilators, such as nitroglycerine^29^, could synergistically contribute to successful hepatocyte transplantation.

In pancreatic islet transplantation, which shares many aspects with hepatocyte transplantation, poor engraftment is strongly associated with an instant blood-mediated inflammatory reaction (IBMIR), characterized by the activation of both coagulation and complement cascades^38,39^. IBMIR has also been reported to occur in hepatocyte transplantation^40^, since hepatocytes as well as pancreatic islets express a substantial amount of tissue factor, which is well recognized as a potent initiator of IBMIR^40,41^. Thus, avoiding IBMIR is crucial for improving hepatocyte engraftment. We have thus far reported that gabexate mesylate^42^, low molecular weight dextran sulfate^43,44^, and C5a inhibitory peptide^45^ can be useful for inhibiting IBMIR. Given that IBMIR cannot theoretically be controlled by isoflurane, the combination between isoflurane and the abovementioned anti-IBMIR treatment would be an attractive approach for successful hepatocyte transplantation.

In the present study, the ischemic regions of the recipient livers in the ISO group tended to be smaller than those in the AW group, although the difference did not reach statistical significance (p = 0.07) (Fig. 6B). Corroborating this finding, the serum levels of AST, ALT, and LDH, which are strongly associated with the damage of hepatocytes^46–48^, were also significantly suppressed in the ISO group in comparison to the AW group (Fig. 2A–C). The ischemic tissues in the recipient livers are indirectly attributed to the occlusion of portal flow by transplanted hepatocytes. However, as shown in Figs. 4B and 5A–D, isoflurane could not contribute to improving the distribution of the hepatocyte grafts in the present study. One possible explanation for this discrepancy is that isoflurane exerts direct protective effects in liver tissue. Of particular interest, Schmidt et al. previously reported that isoflurane efficiently induces heme oxygenase 1, which has a strong cytoprotective effect on hepatocytes, and directly protects liver tissues from liver reperfusion injury^49,50^. In addition, Rao et al. reported that isoflurane directly attenuates liver injury via the restoration of adenosine monophosphate-activated protein kinase/mTOR-mediated hepatocellular autophagy^51^. Taken together, if short-term inhalation of isoflurane is combined with potent vasodilators, the ischemic regions of the recipient livers after hepatocyte transplantation may further decrease and contribute to enhancing hepatocyte engraftment.

Isoflurane has a number of advantages for clinical application. The safety of isoflurane is already established as it is one of the most common inhalation anesthetics and is widely used in daily practice. Isoflurane usually has not been used for hepatocyte transplantation, and it also has not been previously used for the transplantation of several other cell types, including pancreatic islet transplantation, since this aspect has been considered to be one of the most attractive advantages of cell transplantation. However, the present data suggest that isoflurane can be expected to improve the effectiveness of clinical hepatocyte transplantation at least to the level of animal experiments. Thus, it may be justified to replace the advantage of not requiring anesthesia with the use of isoflurane. However, in the close future, isoflurane may be replaced by some reagents that exert similar effects. It is also important to investigate whether venous anesthesia has the same effects as isoflurane, since in some cases although not so common, clinical hepatocyte transplantation has been performed under venous anesthesia.

In conclusion, the present study showed that the anti-inflammatory effects of isoflurane could efficiently contribute to successful hepatocyte engraftment. Thus, isoflurane combined with effective anti-coagulants and/or vasodilators may be a simple yet strong candidate approach to improve the outcomes of clinical hepatocyte transplantation.

## Materials and methods

### Animals

Rat livers were obtained from male inbred F344/NSLc rats (age 10–18 weeks; weight 180–330 g; Japan SLC Inc., Shizuoka, Japan). Analbuminemic rats (age 8–14 weeks; weight 180–280 g) were provided by Prof. Yuji Nishizawa (Asahikawa Medical College) and were bred at Tohoku University. These analbuminemic rats had a syngeneic background to the donor rats. All rats were maintained under a 12-h light/dark cycle with ad libitum access to food and water. All animals were handled according to the Animal Research: Reporting of In Vivo Experiments (ARRIVE) guidelines, the Guide for the Care and Use of Laboratory animals^52^ and the guidelines for animal experiments at Tohoku University. The experimental protocol of the present study (protocol ID: 2020 MdA-149 was approved by the animal experimental committee in the Tohoku University. All surgical procedures were performed under anesthesia, and every effort was made to relieve suffering. In the transplant procedure for the rats in the AW group, we used “Decapicone bags” (Braintree Scientific, Inc., Braintree, MA, USA), which is specifically designed for infusion or administration of several chemicals to the experimental animals, to transplant the hepatocytes with minimal restraint. Prior to initiating transplant experiments, we have fully acclimatized recipient rats to the “Decapicone bags”. Then, in order to relieve suffering from the recipient rats during transplant experiments even under no anesthesia, we have carefully optimized the amount of transplant cells, the volume of transplant solutions, and the duration of transplant procedures, while carefully observing the animal's appearance and body movements suggestive of pains. At the end of the observation period, all animals were euthanized by dissecting the superior vena cava under anesthesia.

### Hepatocyte isolation

Rat hepatocytes were isolated by two-step collagenase perfusion, as previously described^13,14^. First, Ca^2+^-free Hanks’ balanced salt solution (HBSS, Sigma-Aldrich, St. Louis, MO, USA) was perfused thorough the portal vein at a rate of 14 mL/min for 5 min. Second, Ca^2+^-containing HBSS with 0.5 mg/mL of collagenase (Sigma type IV; Sigma-Aldrich) was perfused via the same route at a rate of 14 mL/min for 7 min. The isolated cells were suspended in Dulbecco’s modified Eagle’s medium (Sigma-Aldrich) containing 10% fetal bovine serum (Equitech-Bio Inc., Kerrville, Texas, USA) and 4-(2-hydroxyethyl)-1-piperazineethanesulfonic acid (HEPES) (Gibco, Waltham, MA, USA). The cells were then filtered through a #150 mesh (Ikemoto Scientific Technology, Tokyo, Japan) and purified by gradient centrifugation (50×*g*, 2 min, 4 °C). Density gradient centrifugation (50×*g*, 20 min, 4 °C) with concentrations of Percoll which is density medium for gradient centrifugation (GE Healthcare Biosciences, Pittsburgh, PA, USA) to obtain a highly purified cell population. Hepatocyte viability was evaluated by a trypan blue exclusion assay. For all experiments, we used hepatocytes with a viability exceeding 85%.

### Catheter insertion

**Figure 7:**
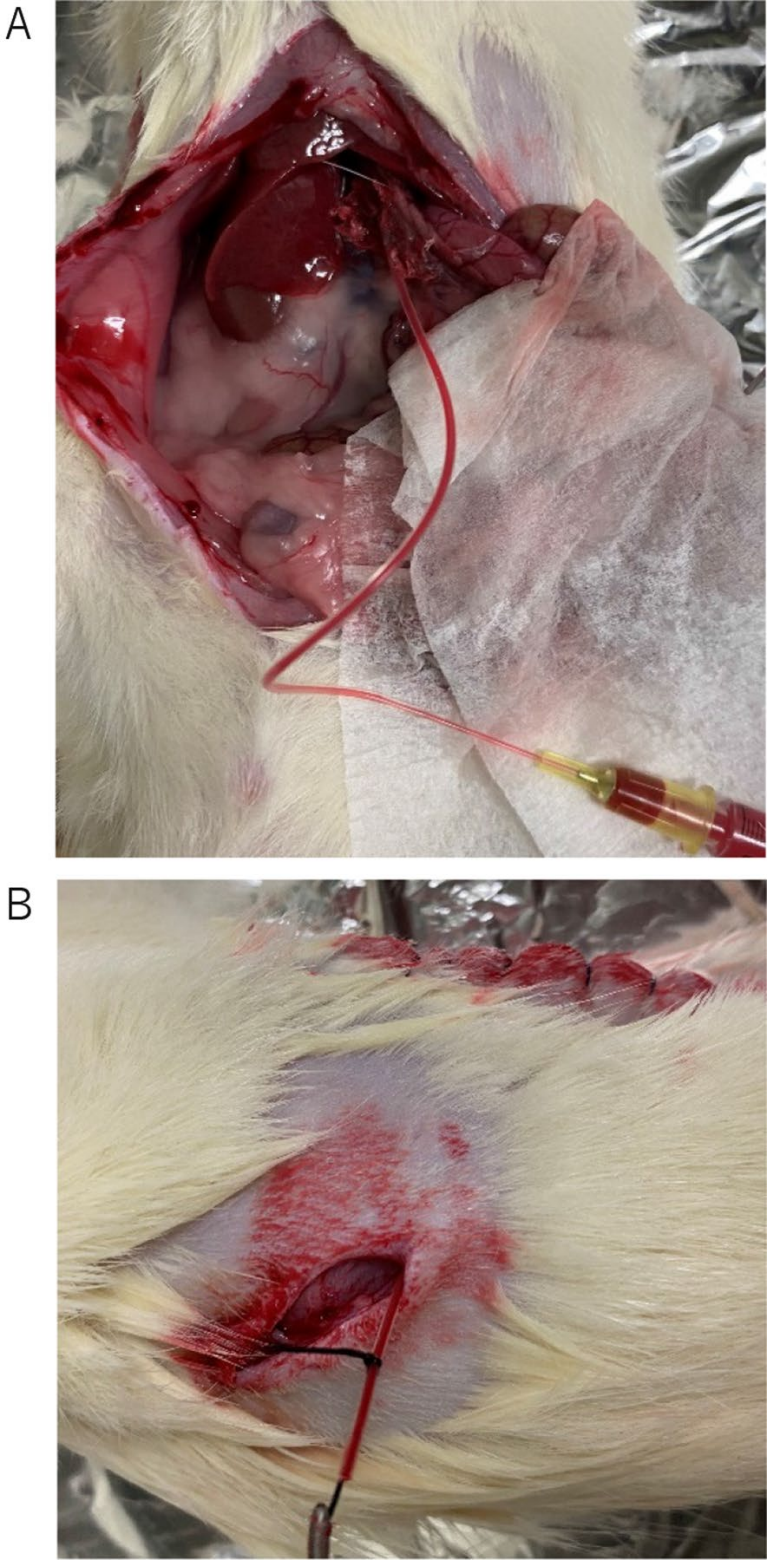
Pictures of catheter insertion. The AW and ISO group were inserted catheter under isoflurane at a concentration of 2.0 L/min with no adjunctive analgesics. (**A**) Under isoflurane anesthesia, a 1 cm skin incision was made in the right lateral abdomen of the recipient rat, then a median abdominal incision was made. A subcutaneous tunnel was created to connect the median abdominal incision to the lateral abdominal incision. (**B**) Under isoflurane anesthesia, the catheter was inserted into the portal vein and fixed using medical Aron Alpha. The catheter was guided out of the abdomen, through the right rectus abdominis muscle and further guided to the lateral abdomen via a subcutaneous tunnel.

The catheter was placed 1 day before transplantation. The portal vein catheter was made by inserting a 5-mm cutting polyimide tube (Furukawa Electric Co., Ltd., Tokyo, Japan) into the 10 cm cutting Silascon tube (Kaneka Corporation, Osaka, Japan). The catheter was flushed with a saline solution containing 1% heparin Na (Mochida Pharmaceutical Co., Ltd. Tokyo, Japan). Small incisions were made in the right lateral abdomen of the recipient rat, then a median abdominal incision was made, and a subcutaneous tunnel to the lateral incision was created. After exposing and puncturing the portal vein, the catheter was fixed at the portal vein using medical grade Aron Alpha A (Sankyo) (Toagosei Co., Ltd., Tokyo, Japan). The catheter was guided out of the abdomen, through the right rectus abdominis muscle and further guided to the lateral abdomen via a subcutaneous tunnel. The abdomen was closed, and the catheter was implanted subcutaneously in the lateral abdomen (Fig. 7A,B).

### Hepatocyte transplantation

Hepatocytes (1.0 × 10^7^) were spontaneously sedimented in the 5 mL macro tube (INA OPTICA, Osaka, Japan) on ice for 30 min to avoid damaging hepatocytes by gradient centrifugation, and the supernatant medium was removed to make pellets. The pellets were slowly (approximately 2 min) injected into the portal vein through the catheter using a 25-G needle with a gastight syringe (Hamilton Company, Reno, NV, USA). The catheter was exposed through the lateral abdominal wound. In the ISO group (n = 10), hepatocyte transplantation was performed under isoflurane at a concentration of 2.0 L/min for 1 h before transplantation, and 2 h after transplantation. In the AW group (n = 10), hepatocyte transplantation was performed without anesthesia. During hepatocyte transplantation the recipient rats did not show any attitudes to escape any signs suggestive of pains.

### Serum albumin

Blood samples were taken from a tail vein at pre-transplantation and every week after transplantation. All transplanted animals were evaluated until 8 weeks after transplantation. The serum albumin levels were quantified using a LBIS rat Albumin ELISA kit (AKRAL-220; Fujifilm Wako Shibayagi, Gumma, Japan).

### Serum AST, ALT, and LDH

In both groups (AW group: n = 10, ISO group: n = 9), blood samples were taken from a tail vein before catheter insertion, at pre-transplantation (time 0), and 1 h, 2 h, 4 h, 8 h, and 24 h after transplantation. The serum AST, ALT, and LDH levels were quantified using Fuji DRI-CHEM 7000 V (Fujifilm Wako Shibayagi).

### Milliplex assay

In both groups (AW group: n = 10, ISO group: n = 9), the IL-1α, IL-1β, IL-2, IL-4, IL-5,IL-6, IL-10, IL-12p70, IL-13, IL-17, IL-18, , Eotaxin, Fractalkine, G-CSF, GM-CSF, GRO/KC, IFN-γ, IP-10, LIX, MCP-1, MIP-1, MIP-2, RANTES, and TNF-α and levels were measured using the Milliplex MAP Rat Cytokine/chemokine Magnetic Bead Panel (Millipore Corporation, Billerica, MA, USA) with a Bioplex 200 system (Bio-Rad, Hercules, CA, USA)^16^. Blood samples were taken from a tail vein before catheter insertion, at pre-transplantation (time 0), and at 1 h, 2 h, 4 h, and 8 h after transplantation.

### Immunohistochemical analyses

In both groups (AW group: n = 6, ISO group: n = 6), recipient livers were retrieved at 24 h after transplantation. Recipient livers were fixed with 4% paraformaldehyde, and embedded in paraffin for immunohistochemical staining. Albumin staining was performed using anti-albumin antibodies (MP Biomedicals, Santa Ana, CA, USA) combined with the VECTASTAIN ABC system (Vector Laboratories, Inc., CA, USA). In both groups (AW group: n = 6, ISO group: n = 6), the number of albumin-positive cells, the location of albumin-positive cells in zones 1, 2, and 3, and the ratio of the ischemic area, which was estimated as the percentage of ischemic tissue in the total liver tissue were calculated by microscopy. In both groups, 6 sections per 1 recipient were examined. When analyzing the number of albumin-positive cells and the location of albumin-positive cells in zones 1, 2, and 3, 10 fields of view were randomly selected and counted. The location of albumin-positive hepatocytes was analyzed in zones 1, 2, and 3 by a pathologist using a blind method.

### Ex vivo imaging

A lipophilic tracer, 1,1-dioctadecyl-3,3,3,3-tetramethylindotricarbocyanineiodide (XenoLight DiR1, Caliper Lifesciences, Hopkinton, MA, USA), was used for labeling hepatocytes^53^. Hepatocytes were labeled according to the manufacturer’s protocol, with slight modifications. The hepatocytes were incubated with phosphate-buffered saline (PBS) containing 25 µg/mL XenoLight DiR (Caliper Lifesciences) for 15 min at 37 °C, and washed twice with PBS buffer, and then resuspended in transplantation medium. DiR-labeling hepatocytes were transplanted into F344 rats (1 × 10^7^ cells/rat). In vivo imaging was performed at 3 h and 24 h after transplantation using an IVIS Spectrum CT imaging system (PerkinElmer, Inc., Waltham MA, USA). DiR fluorescent signals were detected at wavelengths of 745 nm (excitation) and 800 nm (emission). The regions of interest (ROIs) were analyzed, and total quantification of fluorescent signal was quantified using the Living Image software program (PerkinElmer Co., Ltd, Inc.). The ROIs at 24 h were shown as the percentage in comparison to the ROIs at 3 h after transplantation. The rats were then euthanized at the 24 h time point and the liver was harvested for ex vivo imaging. Imaging of the middle and left lobes of the liver was performed to confirm the localization of transplanted hepatocytes.

### Statistical analyses

All values were expressed as the mean ± standard deviation. All statistical analyses were performed using the JMP pro 15 software program (SAS institute Inc., Carry, NC, USA). The serum albumin levels were analyzed by Mann–Whitney *U* test. The serum levels of AST/ALT/LDH were analyzed by a two-way analysis of variance (ANOVA). The number of albumin-positive cells and the ratio of the ischemic area were analyzed using a paired Mann–Whitney *U* test. The serum levels of cytokines were analyzed by Mann–Whitney *U* test. p values of < 0.05 were considered to indicate statistical significance.

## Data Availability

All data generated or analyzed in the present study were included in this published manuscript.

## Abbreviations

ALT: Alanine aminotransferase
ANOVA: Analysis of variance
AST: Aspartate aminotransferase
AW: Awake
EGF: Epidermal growth factor
GRO/KC: Growth related oncogene/keratinocyte-derived cytokine
HBSS: Hanks’ balanced salt solutions
IFN-γ: Interferon-γ
IL-1α: Interleukin-1α
IL-1β: Interleukin-1β
IL-2: Interleukin-2
IL-4: Interleukin-4
IL-5: Interleukin-5
IL-6: Interleukin-6
IL-10: Interleukin-10
IL-12: Interleukin-12
IL-13: Interleukin-13
IL-17: Interleukin-17
IL-18: Interleukin-18
IP-10: Induced protein-10
ISO: Isoflurane
IVIS: In vivo imaging system
LDH: Lactate dehydrogenase
LIX: LPS-induced CXC chemokine
MCP-1: Monocyte chemotactic protein-1
MIP-1α: Macrophage inflammatory protein-1α
MIP-2: Macrophage inflammatory protein-2
RANTES: Regulated upon activation, normal T-cell expressed and secreted.
TNF-α: Tumor necrosis factor-α
VEGF: Vascular endothelial growth factor
